# An ageing study of twenty 18650 lithium-ion Graphite/LFP cells in first and second life use

**DOI:** 10.1038/s41597-025-04712-7

**Published:** 2025-03-06

**Authors:** William Wheeler, Yann Bultel, Pascal Venet, Ali Sari

**Affiliations:** 1 Universite Claude Bernard Lyon 1, Ampère, UMR5005, INSA Lyon, Ecole Centrale de Lyon, CNRS, F-69100 Villeurbanne, France; 2 Univ. Grenoble Alpes, Univ. Savoie Mont Blanc, CNRS, Grenoble INP, LEPMI, F-38000 Grenoble, France

**Keywords:** Electrical and electronic engineering, Batteries

## Abstract

The dataset gives a comprehensive data collection focusing on the ageing of twenty 18650 Graphite/LFP 1.1 Ah commercial cells in first and second life. The ageing from a pristine state up to 40% of capacity loss is uncommon due to the extensive testing time required. Therefore, this new data collection provides insights on long-term ageing of lithium-ion batteries. The initial aim of the study was to comprehend the ageing in first and second-life applications, with a special focus on the sudden acceleration of capacity loss that can occur after a long-term use. The ageing experiment was designed to assess the impact of various test conditions: electric vehicle use, high current charge and discharge, full or partial discharge and a reduced operating voltage window. The cells were characterised every 100 cycles to measure the remaining capacity and the pseudo-open-circuit-voltage. This dataset can help the research community in studying second-life applications or any other study on long-term ageing of Graphite/LFP batteries: new diagnosis and prognosis techniques, AI, battery modelling, and more.

## Background & Summary

The rapid growth of electric vehicles (EV) has led to a significant increase in the demand for lithium-ion batteries which are expected to be used in EV for several years. These batteries are projected to have a State of Health (SoH) ranging from 100% to 70% over their lifetime^1,2^. However, recent data indicates that some batteries may reach the end of their first life sooner, with a State of Health (SoH) higher than 80%^3^. In Europe, recycling EV batteries is mandatory and can provide materials for new batteries^4^. Recycling and producing new batteries can result in higher polluting emissions compared to reuse^5^. However, the remaining capacity of these batteries is sufficient for reuse in stationary applications, such as peak shaving, energy shifting, microgrids, self-consumption, uninterruptible power supply (UPS), vehicle-to-grid/home/building^6,7^. Few studies have been conducted on long-term ageing of Graphite/LFP in first and second life to assess the technical feasibility to reuse EV batteries. To ensure the viability of batteries in second life applications, reliable prognoses of remaining capacity are essential for ensuring economic balance and a reduced environmental footprint.

The collected data was originally motivated to study the ageing of Graphite/LFP cells in order to develop a prognosis method of the remaining SoH and to set up a selection method for a reuse of those batteries in second life applications. This work was conducted in William Wheeler’s PhD thesis, entitled “Reusability of lithium-ion batteries from electric vehicles in second life applications” (manuscript in French)^8^. This study, started in 2019, aimed to understand the sudden and fast decrease in the cell’s remaining capacity, also known as “ageing knee”. This sudden capacity fade needed to be understood in order to predict its apparition, take it into account in an ageing model and finally improve the accuracy of the prediction of the Remaining Useful Life (RuL) of the battery. This information is essential for determining the feasibility of reusing the battery. Research related to this experimental data by Wheeler *et al*. aims to establish a diagnostic method to assess the negative electrode SoH and its impact on the acceleration of capacity fade^9^. The data set was used to establish a diagnosis method using differential analysis such as ICA (Incremental Capacity Analysis) and DVA (Differential Voltage Analysis) on the pseudo-open-circuit voltage (pseudo-OCV) in order to estimate the SoH of the negative electrode^9^. The diagnosis revealed a low decrease of the negative electrode’s SoH in the first life, followed by a faster decrease in the second life. The accelerated capacity loss of the negative electrode is correlated with a significantly faster capacity fade for the cell. A postmortem study on two cells from this test batch confirmed a local loss of graphite in the negative electrode, which reduced the cell cyclability. In contrast, the positive LFP electrode exhibited no defects or capacity loss^10^. A review published on the “ageing knee” may give additional clues about its origin^11^. However, experimental data is required to explain the cause of its apparition.

The study was designed as an accelerated ageing experiment at 50 °C, with intermediate characterization every 100 cycles to assess the impact of various parameters on cell ageing and the development of the “ageing knee.” These parameters include electric vehicle discharge, high current charge and/or discharge, full or partial discharge, and reduced operating voltage window. The cycling ageing study took 24 months, during which the cells were cycled and characterised. Consequently, the cells exhibited a SoH ranging from 50% to 30% by the conclusion of the experiment. The dataset can be used to any other study on battery ageing of Graphite/LFP, in first on in second life: new diagnosis and prognosis methods, artificial intelligence and neural network training, battery and ageing modelling, and more.

## Methods

### Cells and testing equipment

**Fig. 1:**
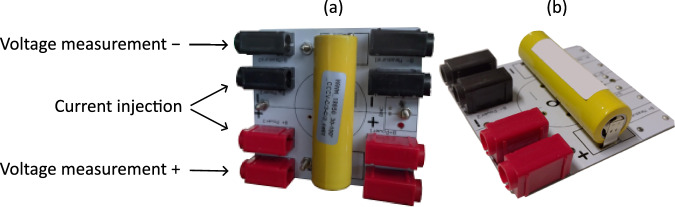
Printed circuit board for 18650 cells. The same printed circuit board can be used in two possible configurations: (**a**) allowing current source in parallel and (**b**) a single current source.

Experiments were conducted on A123 18650 lithium-ion Graphite/LFP with a rated nominal capacity of 1.1 Ah. Additional information from the manufacturer is provided in Table 1. All cells were soldered to a printed circuit board with solder tabs as presented in Fig. 1. The solder tab is connected to the cell with four welding spots. The printed circuit board is equipped with four connectors allowing four-point measurement on the connector tab. Two sockets are employed for current injection, and two sockets are utilized for voltage measurement. It should be noted that some printed circuit boards are equipped with four additional connectors that perform the same function. These supplementary sockets facilitate parallel current, thereby enabling high current experiments.

**Table 1:** Characteristic values of A123 Graphite/LFP 18650 cylindrical cells.

Nominal capacity	1.1 Ah
Nominal voltage	3.2 V
Min/max operating voltage	2 V/3.65 V
Maximum C-rate in charge/discharge	3.6 C/-27 C
Weight	39 g
Diameter	18.2 mm
Length	64.95 mm

Cells were aged using two ARBIN test equipment for ageing and characterisation. Cells were maintained at room temperature (25 °C) for initial and final characterisation. Intermediate characterisation and ageing cycles were performed at 50 °C. Laboratory ovens were used to maintain the cell at 50 °C. It should be noted that the temperature of each cell was not measured due to limitations inherent in the experimental equipment. An Electrochemical Impedance Spectroscopy (EIS) was set up to test each cell at 25 °C prior the experiments and at the end of each ageing test. A Zahner IM6 and PP240 booster, operating in galvanostatic mode with a current amplitude of 200 mA, measurements ranging from 10 mHz to 10 kHz, is used to perform EIS measurements. All equipment specifications are detailed in the Technical Validation section.

### Experiments

Several test conditions were set up in order to assess various applications: different EV uses, stationary applications, or reduced voltage operating window. Table 2 summarised each ageing test case. Each test in conducted on three cells in order to assess the repeatability and a possible variability on ageing results. Test case 4 had only two cells due to laboratory equipment limitations. In this study, cells are used between 2.5 V and a maximum of 3.65 V. The reference test case 1 was designed to emulate a standard electric vehicle use based on a WLTP (Worldwide Harmonised Light vehicles Test Procedure) cycle with a DoD of 70%, a CC-CV charge at C/3 up to 3.65 V with a cutoff current of C/10.

**Table 2:** Summary table of experimental tests conducted on twenty 18650 cells.

Test case	Cell name	Charge type	End of charge cutoff voltage and cutoff C-rate	Charge rate	Discharge type	Discharge rate	DoD
1	Cell 1a	CC-CV	3.65 V – C/10	C/3	WLTP	C/3 on average	70%
	Cell 1b						
	Cell 1c						
2	Cell 2a	CC-CV	3.55 V – C/10	C/3	WLTP	C/3 on average	70%
	Cell 2b						
	Cell 2c						
3	Cell 3a	CC	3.65 V only	C/3	WLTP	C/3 on average	70%
	Cell 3b						
4	Cell 4a	CC-CV	3.65 V – C/10	2 C	WLTP	C/3 on average	70%
	Cell 4b						
	Cell 4c						
5	Cell 5a	CC-CV	3.55 V – C/10	2 C	WLTP	C/3 on average	70%
	Cell 5b						
	Cell 5c						
6	Cell 6a	CC-CV	3.65 V – C/10	2 C	CC	2 C	70%
	Cell 6b						
	Cell 6c						
7	Cell 7a	CC	3.65 V only	C/3	WLTP	C/3 on average	100% (2,5 V)
	Cell 7b						
	Cell 7c						

Each test was performed on three cells, except for test case 3 due to laboratory equipment limitations.

The WLTP cycle simulates the use of an electric vehicle in a 30 minutes current discharge profile. The profile has mainly discharge and rest phases. Few charge phases corresponding to regenerative braking exists. The average discharge current is C/3 with a maximum discharge peak current of 2.7 C and a maximum peak of 1.1 C in regenerative braking. The current profile is repeated until the end-of-discharge condition is reached.

Test case 2 is set in order to assess the impact of lowering the end-of-charge voltage limit. This ageing case is identical to the reference case with a reduced end-of-charge voltage of 0.1 V. Case 3 aims to determine the impact of the end-of-charge protocol under normal use: this case is similar to the reference case but with a CC C/3 load only. Case 4 involve a fast 2 C charge up to 3.65 V and then an end of charge at C/10. The discharge is the same as in case 1. Case 5 is identical to case 4 but with a lower voltage limit set to 3.55 V. Case 6 is similar to case 4 but with a fast 2 C discharge instead of the WLTP discharge. Finally, case 7 have a Depth of Discharge (DoD) of 100%. This case is studied to assess the impact of DoD on the ageing and as a possible cause of the “ageing knee”. The test is carried out with a discharge identical to the reference case 1 until a limit of 2.5 V is reached. In this study, reaching the lower voltage limit of 2.5 V is considered as a full discharge.

### Characterisation and ageing procedure

**Fig. 2:**
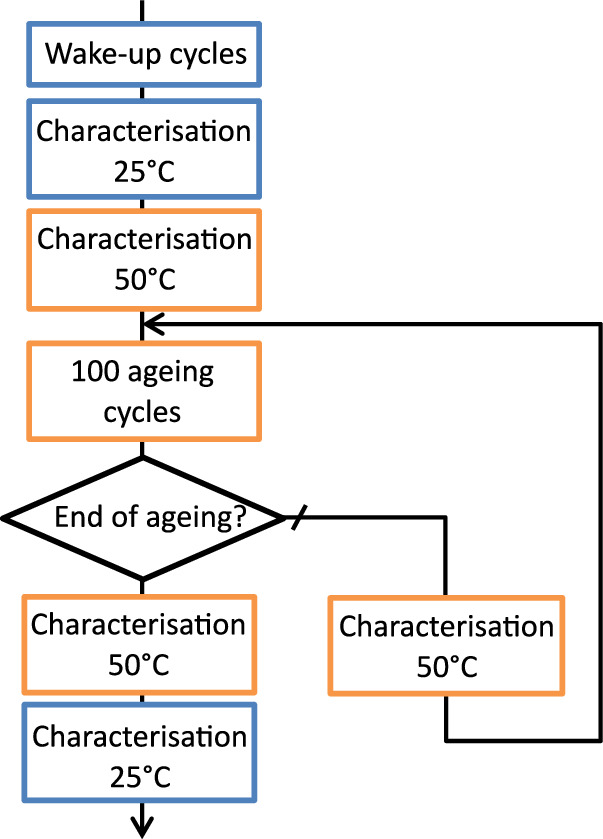
Accelerated ageing protocol flowchart. Steps performed at 25 °C in blue and 50 °C in orange.

The test protocol is identical for all cells. Only the operating parameters in ageing cycles (charge and discharge) are subject to variation, as outlined in the previous section. The test protocol is synthesized in Fig. 2 and includes a cell “wake-up” phase, an initial characterisation at 25 °C and then a characterisation at 50 °C. Then, one hundred ageing cycles at 50 °C followed by an intermediate characterisation are performed. This is repeated until the experiment ends. At the end of the experimental tests, the final characterisations, identical to the initial characterisation, are carried out at 50 °C and then 25 °C. The characterisations conducted at 25°C allow for the assessment of cell capacity and impedance prior to and following the ageing test. The characterisation at 50 °C allow to follow-up the capacity and measure the pseudo-OCV of the cell.

### Wake-up cycles

Prior to the characterisation procedure, “wake-up” cycles are performed to ensure comparable characterisation results for all cells under similar test conditions. The storage time between manufacturing and the start of this study is unknown and can imply uncertainties if know wake-up cycles are performed. The wake-up cycles are identical for all cell ageing experiments. This procedure consists of 25 wake-up cycles. A wake-up cycle is made of 4 steps: a CC discharge at C/3, a rest period of 30 minutes, a CC-CV charge at C/3 up to 3.65 V with a cut-off current of C/10 and a rest period of 30 minutes.

### 25 °C characterisation protocol

**Fig. 3:**
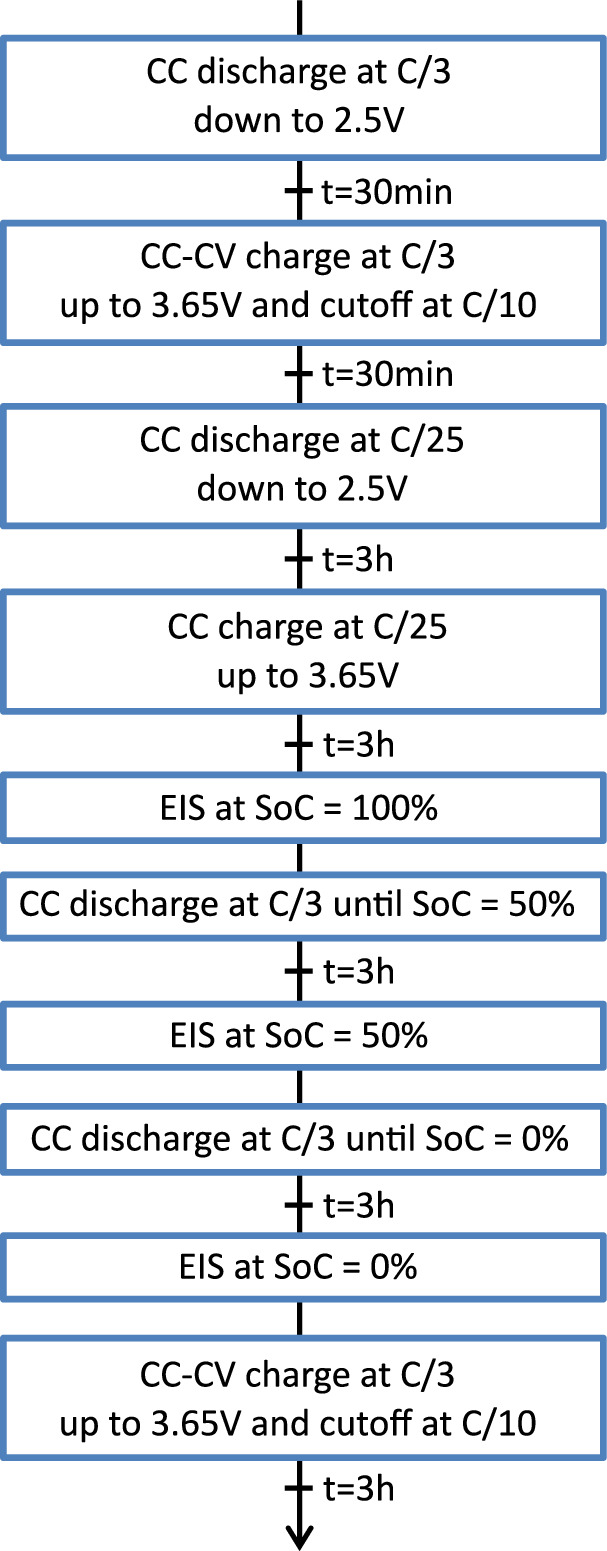
Flowchart of the 25 °C characterisation and intermediate characterisation protocol.

Characterisation at 25 °C is performed as exposed in Fig. 3. The charge and discharge steps at C/3 and C/25 are followed by a cell impedance measurement step at three values of SoC: 100%, 50% and 0%. A rest period of 30 minutes or 3 hours exists between each steps as detailed in Fig. 3. The rest period after the EIS measurement is not specified due to variability in rewiring time.

### 50 °C characterisation protocol

**Fig. 4:**
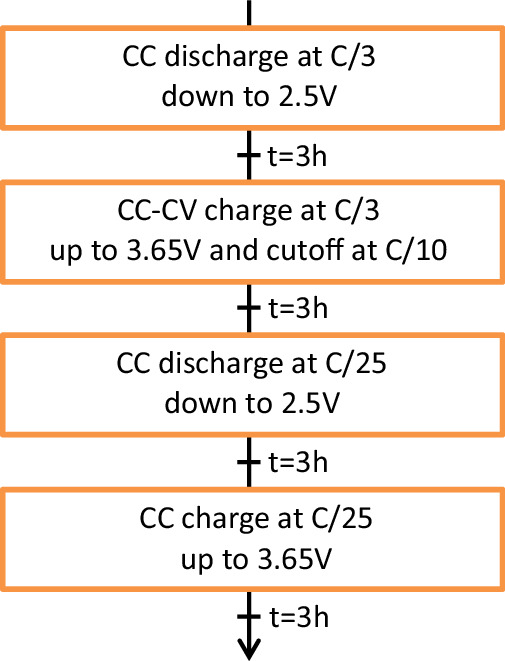
Flowchart of the 50 °C characterisation and intermediate characterisation protocol.

The cells are characterised at 50 °C prior to and following the ageing cycling experiments. An intermediate characterisation with the same steps as the characterisation at 50 °C is performed every 100 ageing cycles. The detailed test steps are illustrated in Fig. 4. The C/3 characterisation allow to follow-up the remaining capacity in the cell and the C/25 characterisation give the pseudo-OCV of the cell.

### Ageing cycles

**Fig. 5:**
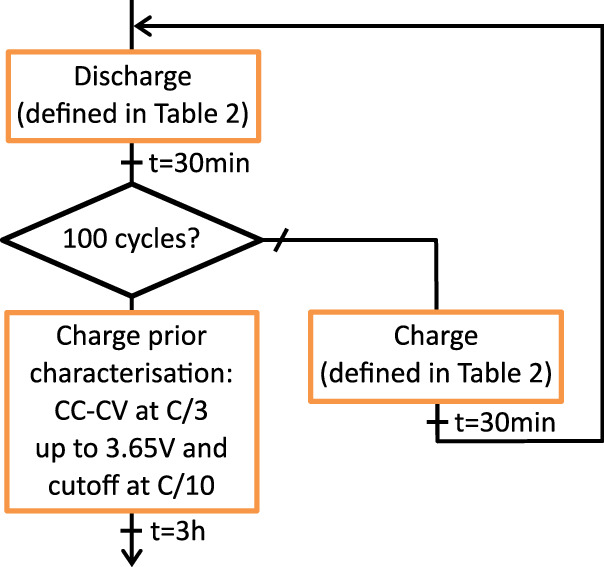
Flowchart for the ageing cycles at 50 °C.

Ageing cycles are carried out with 100 consecutive discharge and charge of the cells. The cycling steps are summarised in Fig. 5. Charging and discharging conditions for each cell are detailed in Table 2. The last charge step is identical for all cells prior the intermediate characterisation step. This final charge is carried out in CC-CV with a C-rate of C/3, a maximum voltage of 3.65 V, and a cut-off current of C/10.

## Data Records

### Data collection architecture

**Fig. 6:**
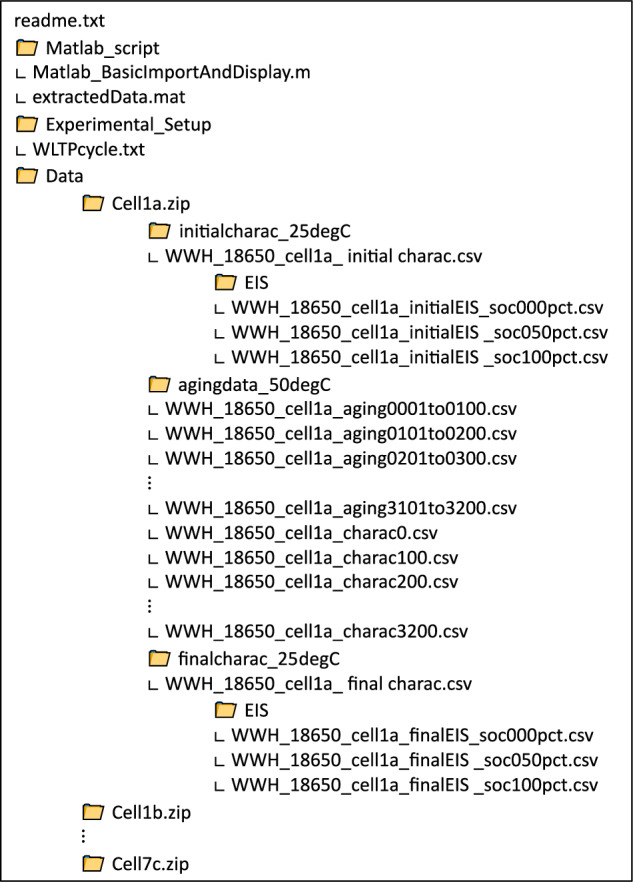
Folder and files architecture of the data collection. Cell 1a is used as an example for all cell folders.

The Data Records is an open data collection available on the French national open data platform “Recherche Data Gouv” with the following name and DOI identifier: “Aging study on twenty A123 18650 Graphite/LFP 1.1 Ah cells”, 10.57745/OLBXKT^12^. This data collection contains a folder for each cell as detailed in Fig. 6. It contains all ageing, characterisation and EIS measurements. An “Experimental_Setup” folder store experimental file such as the current profile for the WLTP discharge cycle. A “Matlab_script” folder contains a script to import and display basic data. Imported data from the script are stored in a.mat file, also available in the “Matlab_script” folder. The script is intended to facilitate and give an example to extract and display the capacity, SoH versus cycles, or EIS. A readme file provides fundamental information about the dataset.

All cell’s files contain the same subfolders: a “initialcharach_25degC” folder containing starting tests at 25 °C (characterisation and EIS files); a “agingdata_50degC” folder containing all tests conducted at 50 °C (ageing and characterisation) and finally a “finalcharac_25degC” folder containing final tests at 25 °C (final characterisation and EIS). In the case of an ageing experiment with a 70% DoD, the discharge capacity is updated based on the last C/3 measurement in charge for the intermediate characterisation. Figure 6 gives an overview of the architecture and the content of the dataset. Figure 6 gives an example of existing files for the cell 1a, cycled from pristine state to 3200 cycles. All files have the same name structure: each file begins with the main author three-letter code, the cell type, the cell name and the data available in the file: ageing from the start cycle number to the final cycle number, a characterisation at a given cycle, or an initial or final EIS at a specified SoC.

### EIS files

Each EIS folder contains three EIS data measurement for the following SoC: 0%, 50% and 100%. The EIS files contain a table header and labels and units for each column of data. Table 3 provides context for each label for EIS files.

**Table 3:** Headers meaning in EIS text files.

Header label	Header meaning	Unit
Number	Line reference number of the data line	
Frequency/Hz	Set frequencies for the measurement	Hz
Impedance R/Ohm	Real part of the impedance for a given frequency	Ω
Impedance I/Ohm	Imaginary part of the impedance for a given frequency	Ω
Significance	Level of noise in each frequency characterisation. The value is determined by the “weighted harmonics autocorrelation” (WHA) algorithm.	
Time/s	The time at the beginning of each frequency measurement from the start of the EIS characterisation (t = 0).	s

### Ageing and characterisation files

Ageing and characterisation files contain labels for each data column in the first line of the file. Each label is detailed in Table 4. Table 4 gives explanations and unit for each label.

**Table 4:** Headers meaning in ageing and characterisation text files.

Header label	Header meaning	Unit
Data_Point	line reference number	
Test_Time	Recorded time from the start of the test file.	s
Step_Time	Recorded time from the start of the test step.	s
Date_Time	Reference number of the step, unique to each step in the experiment. January 1/1900 is serial number 1, and January 1/2008 is serial number 39448 because it is 39 447 days after January 1/1900. Decimal part gives time in a day such as one second has a corresponding value of 1/(24*60*60).	day
Step_Index	Step reference number during the test. Each test step detailed in the Methods section has a unique number: Step reference number during the test. Each test step detailed in the Methods section has a unique number: In ageing files: - 2 or 8: Charge for each ageing cycle (Cf. Tables 2) - 3: rest period - 5: Discharge for each ageing cycle (Cf. Tables 2) - 6: rest period In characterisation files: - 11: C/3 discharge - 12: rest period - 13: C/3 charge - 14: rest period - 16: C/25 discharge - 17: rest period - 18: C/25 charge - 19: rest period	
Cycle_Index	Incremental value giving the cycle number in the ageing file from 1 to 100. In the characterisation file, Cycle_Index take the value 1 for the C/3 charge and discharge and the value 2 for the C/25 charge and discharge.	
Current	Measured current.	A
Voltage	Measured voltage.	V
Charge_Capacity	Charged capacity of a cell in a cycle. Reset to 0 prior to each charge and discharge.	Ah
Discharge_Capacity	Discharged capacity of a cell in a cycle. Reset to 0 prior to each charge and discharge.	Ah
Charge_Energy	Energy in charge mode (current over 0) in a cell in a cycle. Reset to 0 prior to each charge and discharge.	Wh
Discharge_Energy	Energy in discharge mode (current under 0) in a cell in a cycle. Reset to 0 prior to each charge and discharge.	Wh
dV/dt	Differential voltage. Computed as the difference in voltage between the current point and the previous measured point divided by the time between those two measurements.	V.s^−1^

### Supplementary experimental files

The WLTPcycle.txt file is a two columns text file which contains the time in the first column (in second) and the computed current based on a WLTP cycle (in Amps). The WLTP cycle is 1800 seconds long and used in loops until the end of discharge condition is met.

## Technical Validation

### Ageing and characterisation

The ageing study was made possible by the use of two ARBIN instruments dedicated to battery cycling and characterisation. Both equipment has the same precision measurement for voltage and current as detailed in Table 5. All tests are realised using current range 1, except for steps at C/25 which are realised using current range 2.

**Table 5:** Test cyclers characteristics.

Equipment name	Ampère ARBIN 2 A	LEPMI ARBIN 5 A
Manufacturer	ARBIN	ARBIN
Testing equipment	BT-2000	BT-2000
Location	Ampère laboratory	LEPMI laboratory
Number of channels	28	8
Current measurement range 1 and resolution	+/− 2 A (±0.02%)	+/− 5 A (±0.02%)
Current measurement range 2 and resolution	+/− 100 mA (±0.02%)	+/− 100 mA (±0.02%)
Current measurement resolution	16 bit	16 bit
Voltage measurement range and precision	0 to 5 V (±0.02%)	−2 to 10 V (±0.02%)
Voltage measurement resolution	16 bit	16 bit

**Fig. 7:**
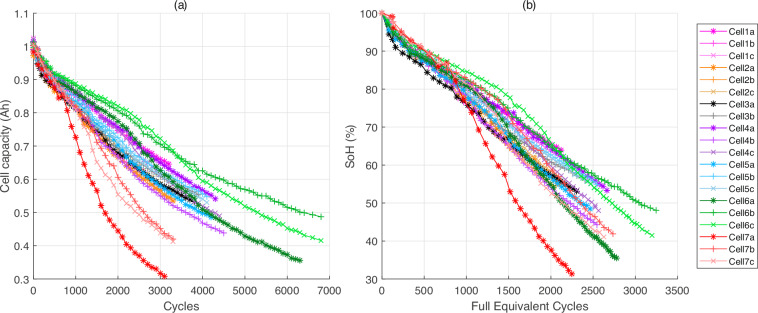
Capacity versus cycle for all tested cells, measured at C/3 in charge in the characterisation. the test was conducted every 100 cycles.

As illustrated in Fig. 7, the capacity of individual cells is plotted against their cycle life. The experimental data indicates that cells within the same test group may possess varying capacities at the conclusion of the experiment, yet they demonstrate a consistent ageing pattern. Furthermore, certain cells exhibit a SoH between 50% and 30% at the end of the 24-month experimental study, attributed to less impactful test conditions. Additionally, there is an acceleration in capacity fade is visible in cells in to groups 6 and 7.

### Electrical impedance spectroscopy

Electrical Impedance Spectroscopy (EIS) was performed with a Zahner IM6 and a PP240 booster, connected with 4 mm plug on the socket soldered to the printed circuit board. Two cables, each made of a pair of two twisted wires, were used respectively for current injection and voltage measurement. Table 6 provides the characteristics of the EIS measurement system.

**Table 6:** EIS equipment characteristics.

Equipment name	Zahner PP240 booster
Manufacturer	Zahner
Testing equipment	PP240
Location	Ampère laboratory
Current measurement range	±1 A
Current Tolerance	±4 mA ±0.2% of reading
Current output resolution	12.5 μA
Voltage measurement range	±5 V
Voltage Tolerance	±1.25 mV ±0.1% of reading
Voltage input resolution	0.596 μV

**Fig. 8:**
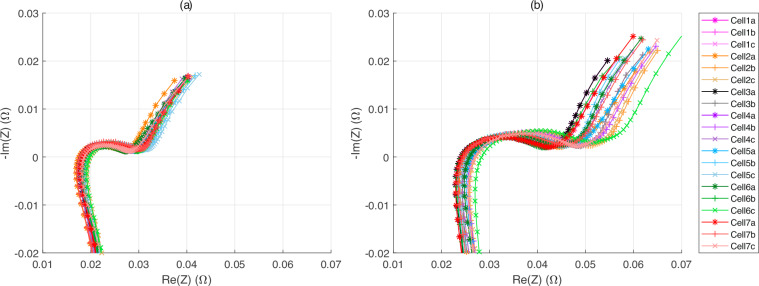
EIS measurements for all cells for the (**a**) initial and (**b**) final characterisation.

As demonstrated in Fig. 8, the impedance for all cells was measured at 0%, 50%, and 100% of SoC prior to and following the ageing experiment. It was observed that all cells exhibited higher impedance at 0% of SoC compared to 50% and 100% of SoC. Furthermore, an increase in both the serial resistance (the impedance value when the imaginary part is zero) and the charge transfer resistance (the length of the real part of the semicircle on the real axis) is observed.

### Laboratory oven

Laboratory ovens were used to ensure that all cells were maintained at the same temperature. A Memmert oven was used in the Ampère laboratory, and a France Etuve oven was used in the LEPMI laboratory. Both ovens were used with forced air circulation, and control measures ensured a maximum variance in air temperature of 1 °C for both ovens.

## Usage Notes

- It is imperative to note that ageing text files can exceed two million lines in length. In such cases, it is strongly recommended to use appropriate software or script commands to efficiently import these large text files.
- It should be noted that ageing and characterisation files may contain additional columns and header labels. However, these columns were not used to record data in the present experiment and, as such, should not be considered.The following labels have been identified as additional and unused labels: “Test_ID”, “Is_FC_Data”, “AC_Impedance”, and “ACI_Phase_Angle”. It is not recommended to use “dV/dt” due to a calculation based on the difference from the current point and the previous available data point. Instead, consider using “Voltage” and “Step_Time” data with filtering and/or resampling and approximate derivatives. “Internal_Resistance” gives the estimated resistance value in some characterisation files, but this option was not available for all test equipments. The value of the cell’s internal resistance is estimated based on a multi-step current of 200 mA, with a step index of 20 and 22 in some characterisation files. However, this data was not used and its accuracy is unknown.
- Five experiments were started prior to the global pandemic COVID19 (SARS-CoV-2). All cells from group 3 and 7 were put on hold during lockdown (cells 3a, 3b, 7a, 7b and 7c). The ageing test was stopped in-between 100 and 200 cycles for all those cells. Therefore, two separated ageing files exist with an intermediary characterisation. The characterisation was made prior restarting the experiment at the temperature of 50 °C, using the 50 °C characterisation procedure. During COVID19 pandemic, cells were stored at room temperature.
- Due to a technical issue, the rest period after the C/3 charge is 30 minutes long instead of 3 hours for some characterisation for cells in group 3 and 7. Cells from group 3 have a 30-minute rest period in the first characterisation file only. Cells from group 7 have a 30-minute rest period for characterisation from cycle 0 to 500. To this date, no impact of the rest period on the characterisation procedure has been identified.
- Due to a technical issue, some data lines are missing in the intermediate characterisation file for the cycle number 100 for cells 1a, 2a, 2c, and 3a.
- Cell 4a is missing two EIS files for the final characterisation at 25 °C at 50% and 0% of SoC due to a manipulation error. Corresponding EIS files exists in the data collection for those two tests, but they do not contain experimental values.

## Data Availability

A MATLAB® script named “Matlab_BasicImportAndDisplay.m” is available in the “Matlab_script” folder of the dataset^12,13^. This script can help new users to access and use the data collection. It is necessary for this script to preserve the database architecture after downloading the dataset. Additionally, all folders must be unzipped. The script also provides examples on how to import data, compute basic indicators such as SoH and create figures. Basic indicators such as cycles and SoH are extracted using this script and saved in the “extractedData.mat” file. Figures 7, 8 in this data descriptor are generated using this MATLAB scripts with MATLAB R2024a.
